# Conceptual foundations of a palliative approach: a knowledge synthesis

**DOI:** 10.1186/s12904-016-0076-9

**Published:** 2016-01-15

**Authors:** Richard Sawatzky, Pat Porterfield, Joyce Lee, Duncan Dixon, Kathleen Lounsbury, Barbara Pesut, Della Roberts, Carolyn Tayler, James Voth, Kelli Stajduhar

**Affiliations:** School of Nursing, Trinity Western University, 7600 Glover Road, Langley, BC V2Y 1Y1 Canada; School of Nursing, University of British Columbia, T-201-2211 Westbrook Mall, Vancouver, BC V6T 2B5 Canada; School of Nursing, University of British Columbia, 1147 Research Road, Kelowna, BC V1V 1V7 Canada; Fraser Health, Delta Hospital, Hospice Palliative Care, 5800 Mountain View Blvd, Delta, BC V4K 3V6 Canada; Fraser Health, Suite 400–Central City Tower, 13450 102nd Avenue, Surrey, BC V3T 0H1 Canada; Intogrey Research and Development Inc., 300-34334 Forrest Terrace, Abbotsford, BC V2S 1G7 Canada; School of Nursing, University of Victoria, PO Box 1700 STN CSC, Victoria, BC V8W 2Y2 Canada

**Keywords:** Palliative approach, Palliative care, Life-limiting illness, Knowledge synthesis

## Abstract

**Background:**

Much of what we understand about the design of healthcare systems to support care of the dying comes from our experiences with providing palliative care for dying cancer patients. It is increasingly recognized that in addition to cancer, high quality end of life care should be an integral part of care that is provided for those with other advancing chronic life-limiting conditions. A “palliative approach” has been articulated as one way of conceptualizing this care. However, there is a lack of conceptual clarity regarding the essential characteristics of a palliative approach to care. The goal of this research was to delineate the key characteristics of a palliative approach found in the empiric literature in order to establish conceptual clarity.

**Methods:**

We conducted a knowledge synthesis of empirical peer-reviewed literature. Search terms pertaining to “palliative care” and “chronic life-limiting conditions” were identified. A comprehensive database search of 11 research databases for the intersection of these terms yielded 190,204 documents. A subsequent computer-assisted approach using statistical predictive classification methods was used to identify relevant documents, resulting in a final yield of 91 studies. Narrative synthesis methods and thematic analysis were used to then identify and conceptualize key characteristics of a palliative approach.

**Results:**

The following three overarching themes were conceptualized to delineate a palliative approach: (1) upstream orientation towards the needs of people who have life-limiting conditions and their families, (2) adaptation of palliative care knowledge and expertise, (3) operationalization of a palliative approach through integration into systems and models of care that do not specialize in palliative care.

**Conclusion:**

Our findings provide much needed conceptual clarity regarding a palliative approach. Such clarity is of fundamental importance for the development of healthcare systems that facilitate the integration of a palliative approach in the care of people who have chronic life-limiting conditions.

## Background

As life expectancy increases, more people are living into old age and dying from serious chronic conditions rather than acute illnesses [1]. Planning care for these and other individuals who are facing life-limiting conditions is vital to a well-managed and person-focused healthcare delivery system. High quality care at the end of life has primarily focused on cancer patients and been delivered by specialist palliative care teams [2]. As such, much of what we understand about the design of healthcare systems to support care of the dying comes from our experiences with caring for dying cancer patients [3, 4]. It is increasingly recognized, however, that in addition to cancer, high quality end of life care should be an integral part of care that is provided for those with other chronic conditions (e.g., heart failure, chronic obstructive pulmonary disease, neurological diseases, renal disease, and dementias) [2, 5–13] and who are being cared for in a variety of settings (e.g., acute care, home care, and residential care) [14–19]. Healthcare managers, policy makers, clinical leaders, and educators are thus faced with questions about how to develop healthcare systems, practice models, and education strategies to best serve this population of people who have advancing chronic life-limiting conditions and their family members.

In 2003, Kristjanson and colleagues articulated a need to understand the potential contribution of palliative care in conditions other than cancer [20]. They articulated a “*palliative approach”* as one way of conceptualizing care for those with advancing chronic illnesses who may not require specialized palliative care services and who would benefit from having their end of life care concerns identified much earlier in the illness trajectory [3, 17, 20]. As part of their cancer control strategy, the World Health Organization (WHO) similarly defined palliative care as an *approach* that is applicable early on in illness trajectories [21]. The Worldwide Palliative Care Alliance (2014) affirmed and adapted the WHO definition emphasizing that palliative care be adopted by all, not just by professionals specializing in palliative care [22].

Others have written about the need to extend palliative care to the care of people with advancing chronic life-limiting conditions, suggesting the need for “early palliative care” [23, 24], “geriatric palliative care” [16, 25], “dementia proofing end of life care” [8], among others. Health services initiatives focusing on a broader implementation of palliative care principles included the Australian Palliative Residential Aged Care (APRAC) Project and the Program of Experience in the Palliative Approach funded by the Australian Government [26] as well as the Gold Standards Framework [27] that was initially developed to enhance primary palliative care in the United Kingdom. Such initiatives provided an important impetus for research on a palliative approach and have contributed to the development of tools and guidelines for improved end of life care (e.g., Guidelines for a Palliative Approach in Residential Aged Care [28]) that would better guide clinician education and care provision. Indeed, in the last decade there has been a proliferation of research focused on the palliative care needs of people with advancing chronic and life-limiting conditions [29–33]. Inasmuch as an articulation of the need for a broader approach has been expressed and evidence suggests that such an approach may have positive outcomes for people who are on a progressive life-limiting trajectory [23, 34], there is a lack of conceptual clarity regarding the essential characteristics of what a palliative approach entails.

As part of a program of research to address how and in which contexts a palliative approach can better meet the needs of people with chronic life-limiting conditions and their family members, the iPANEL team (Initiative for a Palliative Approach in Nursing: Evidence and Leadership – www.ipanel.ca [35]) is pursuing several primary research and integrated knowledge translation activities that address research questions relevant to a palliative approach [4, 36]. A core focus of our applied health services research program involves an overarching knowledge synthesis regarding healthcare systems policy, education, and practice initiatives for a palliative approach. In this paper we report on one aspect of this knowledge synthesis focusing specifically on delineating key characteristics of a palliative approach that are found in the empiric literature in order to establish conceptual clarity. Our goal is to provide guidance to healthcare professionals wishing to integrate a palliative approach into their practice, health systems managers and decision makers interested in integrating a palliative approach into their care delivery models, and researchers needing to articulate core conceptual features of a palliative approach to guide their studies.

## Methods

We conducted a comprehensive mixed-methods knowledge synthesis of empirical peer-reviewed literature, including quantitative and qualitative research and reviews. Established knowledge synthesis procedures were implemented to search for relevant sources, extract relevant information from each source, and conduct a synthesis [37–40].

### Search and selection of articles

**Fig. 1:**
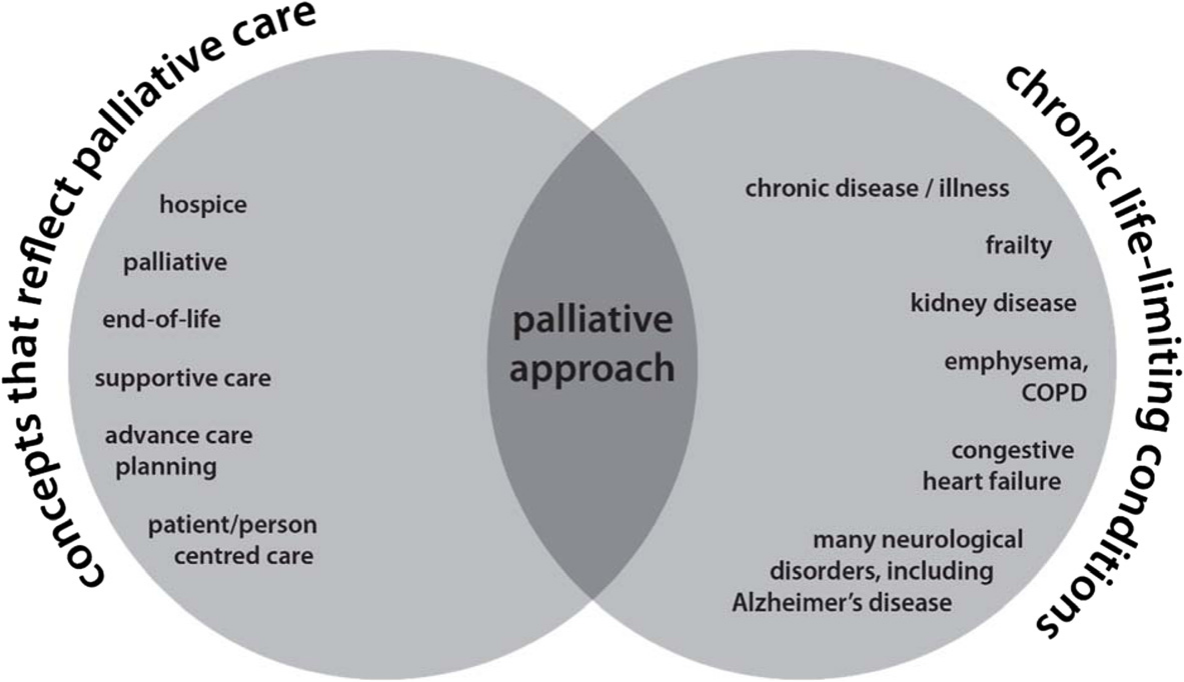
Identifying a palliative approach in the literature

The search strategy was designed to identify articles of potential relevance to a palliative approach, even though the term “palliative approach” may not have been used. In other words, we sought to identify those articles that address the integration of hospice, palliative, or end of life care principles and practices for people with chronic life-limiting conditions. Accordingly, the following search terms were delineated to focus on the intersection of the following two domains: (a) concepts associated with palliative care (including hospice care, comfort care, end of life care, etc.) and (b) concepts reflective of chronic life-limiting conditions (including a selection of the most common chronic conditions) (see Fig. 1). Search terms for each domain were identified based on a preliminary scoping search and in consultation with the iPANEL team and other expert researchers and clinicians.

Reed and Baxter’s [41] recommendations for searching reference databases were followed. Separate search strategies were designed by combining keywords and applicable subject headings (where a thesaurus was available) for each of the following databases: Ageline, Biomedical Reference Collection: Comprehensive, CINAHL, Cochrane Database of Systematic Reviews, Embase, Healthsource Nursing/Academic, Medline, ProQuest Dissertations & Theses, PsycINFO, and Web of Science. The searches were limited to English language articles published between January 1 1990 and December 31 2011. The search results were subsequently imported into a database and duplicates were removed. The final combined searches yielded 190,204 citations.

**Fig. 2:**
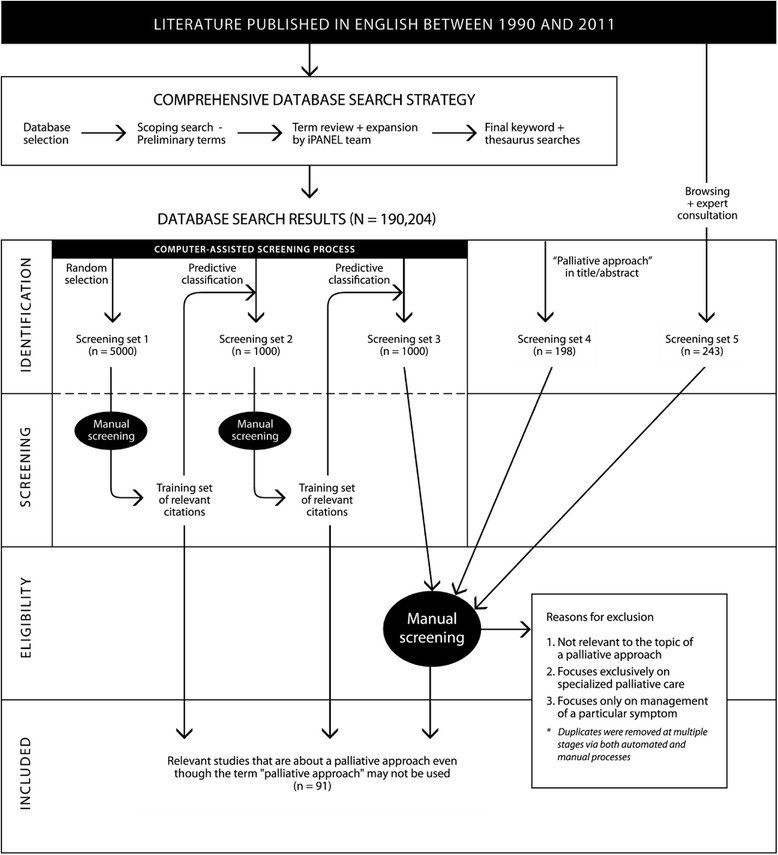
Literature search and selection strategy

Because a palliative approach is often not explicitly mentioned or referred to in a consistent way, it was impossible to further delineate the search without inadvertently excluding potentially relevant documents. We therefore designed and implemented a computer-assisted approach using a statistical predictive classification method [42–45] to probabilistically identify citations that are likely to be relevant. Terminology (in this case, words) was extracted from a *“computer training set”* of citations that were manually screened and classified as either being relevant or not relevant, and was then used to progressively refine the predictive classification model (see Fig. 2). If an unclassified citation contained similar terminology to those manually classified as relevant in the training sets, then it was considered more likely to also be relevant. Conversely, if an unclassified citation contained similar terminology to those citations that were manually classified as irrelevant, then it was considered more likely to not be relevant.

To begin, a random selection of 5,000 citations (“screening set 1”) was drawn from the complete search result set and was manually screened; this created the first computer training set. The predictive classification model was generated using this training set and used to identify another set of 1,000 citations that were likely to be relevant (“screening set 2”) from the complete search result set. These citations were subsequently manually screened and added to the training set to refine the predictive classification model. The model was reapplied to identify another set of 1,000 citations (“screening set 3”), which was then manually screened. In addition to the articles identified through predictive classification, 198 articles that included the term “palliative approach” in the title or abstract (“screening set 4”), and 243 articles recommended as being relevant to a palliative approach by the iPANEL team members and experts in the field (“screening set 5”), were manually screened. Through this process, a total of 338 relevant documents (including both research and non-research based articles) were identified. Of these, 91 articles reported on primary research and literature reviews.

The following inclusion criterion was used to identify relevant documents: the document must be clearly about or focused on a palliative approach, defined as the integration of palliative care principles into healthcare settings by professionals who do not specialize in palliative care, even though the term “palliative approach” may not be explicitly mentioned. Documents that focused predominantly on specialized palliative care (provided by specialized palliative care professionals) or on the management of one particular symptom (rather than overall care of a person who has a life-limiting condition) were excluded. In addition, only articles reporting on primary research and literature reviews were included. A consensus-based approach was used to double-screen the articles. Articles that were inconsistently classified by different screeners as both relevant and not-relevant were reviewed by two additional team members to establish consensus. The Evidence for Policy and Practice Information and Co-ordinating Centre Reviewer software (EPPI-Reviewers version 4.1) [46] was used to combine all documents into a common database and extract relevant information using a data extraction codebook designed for this review.

### Analysis methods

We used narrative synthesis methods and thematic analysis to identify and conceptualize essential characteristics of a palliative approach [37–40]. A data extraction form was developed and applied in EPPI-Reviewer to initially classify articles in terms of their focus on particular disease populations, healthcare sectors, and a range of study characteristics (e.g., study design, study methods, sample characteristics). We subsequently compared the patient populations and the nature of the care provided across different conditions and contexts of healthcare delivery. Following general guidelines to narrative synthesis described by Pope and Popay [40], thematic analysis was applied by first extracting and organizing verbatim quotes and paraphrased text pertaining to a palliative approach into categories that represent common characteristics of a palliative approach. Higher level abstract themes were iteratively inferred by comparing and contrasting similarities and differences across categories and classifications as the basis for arriving at a conceptualization of key characteristics of a palliative approach that applies across disease groupings and contexts of care.

### Limitations

Although our search and analysis methods were comprehensive, there are several limitations that need to be taken into account when considering the findings. First, considering the scope of literature relevant to our topic, it was not feasible to conduct a synthesis of all relevant literature. We therefore used a probabilistic computer-assisted approach to identify studies that were likely to be relevant. In addition, our analysis was limited to research articles. Nonetheless, we have found that our synthesis findings are consistent with studies and other sources that were not identified through this process [6, 7, 12, 47–49], and are therefore confident that inclusion of these additional sources would not substantially change the conceptualization of a palliative approach that arose from our synthesis. Second, the method of analysis is inherently interpretive in nature. Well-established qualitative knowledge synthesis methods were followed to reach a higher level of abstraction than what was explicitly stated in any individual article. Third, it is important to keep in mind that only articles written in English were considered. Consequently, studies arising from non-English speaking countries will be underrepresented.

## Results and discussion

A description of the articles included in the analysis is presented in Tables 1 and 2. The included quantitative and qualitative studies are predominantly based on study populations from the US and UK; nine studies are from Canada (Table 1). The studies cover a range of health conditions and healthcare sectors (Table 2). Most of the studies address multiple chronic conditions (32 %), followed by cancer (14 %), and dementia (10 %) and focus on hospital, residential, and home/community sectors.

**Table 1:** Description of relevant studies (*n* = 91)

Study type	No.	Study method	No.	Country	No.
Published/Presented paper	88	Quantitative	33	Australia	9
Thesis/Dissertation	3	Qualitative	33	Canada	9
		Mixed methods	16	UK	28
		Knowledge synthesis	9	US	35
				Other^a^	10

^a^China (1), Germany (2) Global (2), Israel (1), Italy (1), Netherlands (2), Sweden (1)

**Table 2:** Classification of relevant studies by health conditions and healthcare sectors (*n* = 91)

		# Articles by healthcare sector^a^			
Health conditions	# Articles	*Hospital*	*Residential*	*Home and community*	*Other* ^*b*^
Multiple chronic diseases	29	8	8	10	4
Cancer	13	6	2	6	3
Dementia	9	1	8	2	1
COPD	4	1	0	2	1
Neurological diseases	4	2	1	1	2
AIDs	2	0	0	1	1
Renal disease	2	2	0	1	0
Frail elderly	4	0	2	2	0
Congestive heart failure	2	1	0	1	0
Other	1	0	1	0	0
Not specified	21	12	5	3	4
Total	91	33	27	29	16

^a^Coding is not mutually exclusive as some studies addressed multiple healthcare sectors

^b^hospice care (3 studies), unclear/unspecified (10 studies), education (3 studies)

### A palliative approach to care

A palliative approach builds on many of the key principles foundational to *specialized palliative care services*. Like specialized palliative care, a palliative approach emphasizes patient- and family-centered care that focuses on the person and not just the disease, where quality of life is seen as the primary goal [5, 6, 17, e.g. 50]. Literature on a palliative approach highlights the importance of therapeutic relationships between providers and the patient and family with an emphasis on building partnerships to enhance care quality [9, 51]. Clear communication throughout the illness trajectory is stressed in the literature as significant, particularly in relation to conversations about advance care plans, goals of care, “breaking bad news”, and shifts in the management of the disease process or the plan of care. A palliative approach is based on the foundations of palliative care in its emphasis on careful assessment and management of disease-associated symptoms and on the importance of compassionate and skilled care for patients who are imminently dying.

Findings from our narrative synthesis and thematic analysis suggest that there are key distinctions to be made between a palliative approach and the way in which specialized palliative care has conventionally been enacted as a healthcare service. Although the notion of a palliative approach has been represented and taken up in different ways, there is an emerging understanding that, broadly conceptualized, a palliative approach involves adopting the foundational principles of palliative care, adapting the palliative care knowledge and expertise to the illness trajectories of people with chronic life-limiting conditions, and embedding this adapted knowledge and expertise “upstream” into the delivery of care across different healthcare sectors and professions. Consistent with this conceptualization, our synthesis of the literature resulted in three themes that represent essential characteristics of a palliative approach: (a) an upstream orientation to care, (b) adaptation of palliative care knowledge and expertise, and (c) operationalization of a palliative approach through integration and contextualization within healthcare systems. These themes are further discussed below and are illustrated by examples of citations and quotes in Table 3.

**Table 3:** Thematic representation of key characteristics of a palliative approach

Thematic categories with citations	Illustrative quotations
Theme 1: Upstream orientation to care	
Recognition of diverse illness trajectories [2, 8–10, 13, 15, 16, 19, 23, 25, 30, 34, 51–57, 59, 61–63, 65, 67–98]	*“Neurological diseases present and progress with great clinical variation. In contrast to patients in many other areas in medicine, neurological patients frequently live many years while developing cumulative physical and cognitive disabilities. As a result, patients living with neurological disease cope with decreasing quality of life before reaching the terminal stage of their illness”* [2]. *“The illness trajectory of patients dying with chronic kidney disease differs from that of patients with cancer. There are also unique end of life issues, such as the withdrawal of dialysis, that are specific to these patients. The existence of different pathways to death has important implications for health care delivery; end of life issues that are important to chronic kidney disease patients may differ from patients dying from other illnesses”* [10].
Importance of identifying where people are on their illness trajectory [2, 8–10, 13, 15, 16, 19, 23, 25, 30, 34, 51–57, 59–61, 63, 64, 67–71, 74–79, 81, 82, 84, 85, 87–91, 93, 94, 96–103]	*“Communication of prognosis and discussions related to planning for future death are lacking in the routine care of CKD patients…. Answering yes to a simple question “Would you be surprised if this patient died with the next year?” should also prompt the nephrology team to initiate these discussions”* [10].
The challenge of prognostication [2, 9, 10, 13, 16, 23, 34, 51–57, 59, 60, 63, 65, 67, 68, 70, 72, 75–78, 84–87, 90, 92, 95, 97, 101, 102, 104–110]	*“…determining the trajectory of an individual’s illness is an inaccurate science, thereby making lane changes difficult…While nurses in the study experienced physicians as “waffling” when they were not specific about prognostication, the literature suggests that, because of the unreliability of prognostic judgements, physicians may opt to provide a range of survival estimates. What makes prognostication even more difficult within the context of acute medical units is the range of patients that are cared for and the manner in which their symptoms present”* [102].
Theme 2: Adaptation of palliative care knowledge and expertise	
Adaptation throughout all aspects of the care process, including: a) individualized assessment of care needs [2, 8–10, 13, 16, 19, 23, 25, 27, 30, 34, 51–57, 59–65, 67–73, 75–81, 83–85, 87–101, 103, 104, 106–108, 110–121]	*“If end of life care does not take into account the unique circumstances and needs of people with dementia, it is likely to fail them”* [8]. *“Medical visits should begin with an assessment of the patient’s agenda and issues, including immediate concerns and threats to quality of life”* [92].
b) symptom management [2, 8–10, 13, 16, 19, 23, 25, 27, 34, 51–57, 59, 61–63, 67–70, 72–74, 77–82, 85–88, 90, 91, 93, 94, 96–99, 101, 103, 105, 106, 108, 110, 112–116, 118, 120–126]	*“Effective symptom management may require multiple therapeutic components (pharmacological and nonpharmacological) or involvement of multiple disciplines. This approach is a foundation of geriatrics and is based on the demonstrated effectiveness of programs of care that provide assessment and targeted remediation of multiple factors in preventing falls and delirium”* [8].
c) communication and care planning (goals of care, advance care planning, anticipatory care planning) [8–10, 13, 16, 19, 23, 25, 27, 30, 34, 51–57, 59–63, 65, 67–105, 107, 109–120, 122, 123, 125, 127]	*“Checking the patient’s and family members’ understanding and goals should be an ongoing process during the course of the disease and will facilitate the process of advance care planning”* [51]. *“Important factors that determine when the patient and family are ready to discuss end of life issues include coping skills, depression and anxiety, cultural issues, use of functional assistive devices, and physiologic status, among others”* [13].
Patient, family, and caregivers as partners in care [8–10, 13, 16, 19, 25, 30, 34, 51–57, 59–65, 67–70, 72–82, 84–87, 89–95, 97, 98, 100, 102, 104–108, 111–116, 118–123, 126, 128]	*“Symptom self-management support involves helping patients and their families acquire the skills and confidence to manage their illness. Education and support can also help patients and families weather the variable, day-to-day […] nature of common symptoms and their effect on ADLs as well as to prepare for an emergency”* [16]. *“To minimize hospitalizations, she is willing to participate in the distance-monitoring program offered by the local home health agency. The nurse will monitor daily weights, blood pressure, and pulse, and communicate changes to the NP so the medication regimen can be promptly adjusted. The agency also provides case management. In this way, Mrs. C’s complex needs can be continuously assessed and promptly addressed. The NP also offers a referral to a licensed clinical social worker who can provide support and counseling to her and her family”* [54]. *“This project aims to develop a model of integrated care involving partnership between older adults, their carers, and health professionals though a system of mentorship and information technology (IT) provision. The intent is to assist older people with chronic respiratory conditions to enjoy enhanced self-efficacy and quality of life by interactions with a community nurse mentor, coupled with the use of a technology-supported system of self-management”* [9].
Theme 3: Operationalization of a palliative approach through integration and contextualization within healthcare systems	
Models/systems of care for a palliative approach: a) “early” palliative care [23, 52, 57]	*“A palliative approach, also referred to as simultaneous care, acknowledges the likelihood of gradual transition, emphasizing quality of life considerations during the active treatment phase. It recognizes that treatment goals will evolve from seeking a cure, to control of disease and complications, maintaining physical functioning and quality of life, and ultimately to symptom control …. Early introduction of the palliative care health professionals as part of the multi-disciplinary treatment team can facilitate the transition from curative to palliative treatment. It is important that the palliative care professionals are seen as an integral part of the treatment team, which will enhance the sense of continuity of care and allay any fears of abandonment”* [57].
b) integration into generalist practice [15, 19, 27, 34, 61–64, 78, 83, 85, 88, 89, 92, 94, 99–101, 103, 107, 115, 117–119, 126, 129]	*“For nurses, the GSF appeared to provide structure, authority and permission to arrange both formal meetings with general practitioners and informal communication opportunities that facilitated the achievement of professional and patient care objectives… Systems were described to discuss patients within the primary health care team to ensure that people were aware of patients and that they were referred at an ‘appropriate’ time”* [103].
c) disease/condition-specific models for care delivery [2, 8–10, 13, 16, 51, 53, 55, 56, 59, 65, 68, 91, 93, 96, 97, 111]	*“The study’s primary finding was that there is a need to ‘dementia proof’ end of life care for people with dementia. If end of life care does not take into account the unique circumstances and needs of people with dementia, it is likely to fail them. This requires service providers and care professionals to ensure that the environments in which people live and die – be they at home, in a care home, in NHS continuing care or in a general hospital – do three things: use knowledge of dementia to identify and respond to physical care needs; go beyond task focused care; and prioritise planning and communication with the family”* [8]. *“Patients with heart failure need comprehensive palliation, regardless of disease stage or need for aggressive therapy. Collaborative, interdisciplinary care provided by the joint teams have benefited patients, families and staff by attending to symptoms, establishing goals of care, and planning for life outside the hospital”* [96]. *“It seems equally evident that the respiratory community has much to learn from the palliative care community about the breadth of service provision required. We suggest that only by working together and educating each other can we provide the expertise to respond to the above issues and support patients/carers across the respiratory–palliative care spectrum”* [53].

### Theme 1: upstream orientation to care

A key characteristic of a palliative approach is an upstream orientation that ensures that the needs of patients and families are addressed early on and throughout the illness trajectory of people who have chronic life-limiting conditions. The importance of recognizing the life-limiting nature of many chronic conditions that will ultimately lead to death, and the need to consider principles of palliative care early on in the illness trajectory, even as soon as the time of diagnosis, has been noted in several studies as well as in an expert review [2, 15, 23, 52]. Although this upstream orientation applies to many potential chronic life-limiting conditions, it has thus far been predominantly articulated in contexts of care for people who have chronic obstructive pulmonary disease (COPD), congestive heart failure (CHF), renal disease, neurological diseases (in particular dementias and amyotrophic lateral sclerosis), and general frailty [2, 9, 10, 16, 53–56]. Across domains of chronic disease literature, experts consistently identify two conditions required of care providers to achieve an upstream orientation: (1) recognition and understanding of different chronic illness trajectories, and (2) identification of where people are on those trajectories (see citations and quotes in Table 3). People on those trajectories require care that is oriented by the knowledge that their illness is life-limiting.

Lynn’s [49] characterization of chronic life-limiting illness trajectories is widely referenced. Three dominant trajectories of functional decline and wellbeing are described: (1) the “cancer” trajectory, characterized by a long period of relatively good function with a sudden decline leading to death; (2) the “organ system failure” trajectory that involves an ongoing gradual decline with periods of severe exacerbation with death often coming rather suddenly (e.g., COPD, CHF, and kidney diseases); and (3) the “dementia/frailty” trajectory associated with a prolonged decline, relatively low function, and slow deterioration. Palliative care has predominantly been developed in relation to the first trajectory, which, originally, was primarily applicable to people with cancer who no longer receive curative treatments. Within the cancer illness trajectory, palliation has been considered a distinct and relatively predictable phase occurring near the end of life. It should be noted, however, that cancer trajectories themselves are changing and that certain forms of cancer now follow trajectories that are similar to those of other chronic conditions [57].

Although there are some illnesses with a relatively predictable end-stage, it is increasingly recognized that the cancer trajectory is not representative of the course of many chronic life-limiting conditions. The above second and third trajectories described by Lynn [49] are distinguished from the cancer trajectory in that they have an element of uncertainty regarding illness progression and estimation of when death will occur. The studies in our synthesis consistently emphasize that the uncertainty associated with these trajectories and the corresponding challenge of prognostication necessitates an upstream orientation to emerging end of life care needs (see citations and quotes in Table 3). In addition, attending to the progressive nature of the illness, while at the same time recognizing that these people may live for a long period of time, requires concurrent chronic disease management. Thus, a palliative approach is achieved through the blending of palliative care and chronic disease management with a focus on quality of life as the primary goal [9].

Significant advances have been made in finding ways to identify people who are on advancing illness trajectories and in need of a palliative approach [54] as people on these chronic illness trajectories were formerly considered in need of end of life care only during the last weeks and days of life. An upstream orientation to care facilitates proactive care planning [15], advance care planning [2, 19, 51], goals of care conversations [57], and the ability for patients and family members to be part of decision making regarding their care [2, 9]. Identification criteria and tools have been developed to reveal patient needs that arise along the chronic illness trajectories and to identify people at high risk of dying. An example includes the widely-cited prognostic indicators of the UK Gold Standards Framework (GSF) [58], which consists of three identifying triggers: (1) the “surprise question” (“would you be surprised if the patient were to die in the next few months, weeks, days”); (2) general indicators of decline; and (3) specific clinical indicators for particular conditions (e.g., cancer, COPD, heart diseases, renal diseases, neurological conditions, stroke, dementia, frailty). An important dimension of identification is that it is an ongoing process of identifying end of life care needs as they emerge throughout the illness trajectories.

### Theme 2: adaptation of palliative care knowledge and expertise

Although a palliative approach builds on the knowledge and expertise of palliative care, it is also apparent that it needs to be adapted to the particular care needs of people with chronic life-limiting conditions. A palliative approach is not simply *applying* knowledge and expertise from palliative care to practice; it requires *adaptation* to different patient populations and their unique disease profiles [2]. This is particularly important because of the uncertainty related to prognosis and the course of illness for people who have life-limiting chronic conditions. Unlike some cancer illness trajectories where time until death is relatively predictable, chronic conditions are marked by exacerbations of the disease and periods of stability. The individualized assessment of care needs and the corresponding treatment decisions must therefore be based on the recognition that death is inevitable but may take a long time to occur. This has particular implications for knowledge and expertise regarding symptom management, communication, and partnerships with patients and families, as is illustrated in Table 3.

While the preponderance of palliative care knowledge on symptom management is based on populations of people with cancer at end of life, other illnesses also have high symptom burden. Studies of patients with COPD, for instance, reveal variability in how symptoms such as breathlessness and dyspnea are treated because treatment approaches may differ depending on survival predictions [53]. Adaptations of palliative care knowledge about symptom management are also required because of differences in the pathophysiology of chronic conditions. For example, although patients with advanced renal disease have pain control needs, conventional approaches to pain management in cancer, consisting of high dose opioids, will need to be adapted for renal patients to avoid accumulation of toxic metabolites [59].

In addition to the need for adaptations in symptom management, studies suggest that adaptations to the ways in which we communicate with patients and family members also are required because of the uncertainty of the illness trajectory. Although the possibility of death is generally recognized in the care of cancer patients, patients with chronic life-limiting conditions are sometimes not aware of the extent of their disease or even that their illness is progressive and life-limiting [10]. Conversations related to care planning that are more typically associated with specialized palliative care (e.g., goals of care, advance care planning, anticipatory care planning, see Table 3) may require adaptations that are sensitive to the needs of those patients who have not yet identified themselves as a person with an illness that will eventually lead to death. Studies with chronically ill people and their care providers demonstrate that some view palliative care as giving up on treatment, which dispels hope [10, 57, 60]. As such, adaptations in communication strategies and the timing of conversations about sensitive subjects, such as end of life closure, are needed. The trajectory of heart failure is one example. Characterized by repeated near death exacerbations of illness that are treated with combinations of pharmacological and device therapy, heart failure patients experience living in between hope for continued quality of life and the possibility of imminent death due to cardiac arrest. As such, communication strategies require adaptation to guide providers in talking with patients and families who are living in between the hope of remission of symptoms and the possibility that the patient might die [54].

The literature describing a palliative approach also draws heavily on the fundamental principle of partnership in palliative care that emphasizes the patient and family as the unit and focus of care. In palliative care, the partnership with patients and families is predominantly focused on end of life care needs and, because of the advanced stage of the disease and the burden of illness, involves an active role of the healthcare provider in meeting those needs on behalf of patients and families. The palliative approach necessitates adapting the partnerships based on the changing needs of patients and families in relation to their illness trajectories. Drawing from knowledge that originated from chronic disease self-management, and because the burden of illness initially tends to be less, there is a greater emphasis on engaging patients and families in self-management strategies that include end of life considerations earlier on in the illness trajectory. This self-management philosophy is exemplified in the notion of a palliative approach incorporated within pulmonary rehabilitation [53] or projects such as The Pathways Home Project [9], as is quoted in Table 3. Self-management is consistent with the goal of improving quality of life, which for many patients and families means maximizing function to enable them to do what is important and meaningful for them. As the patient and family approaches death, the nature of the partnering shifts with healthcare providers taking a more active role in “doing for” within the context of compassionate care, while at the same time respecting and being sensitive to the self-management strategies that have been adopted by patients with chronic conditions and their family members.

### Theme 3: operationalization of a palliative approach through integration and contextualization within healthcare systems

Delivering a palliative approach early on in the illness trajectories necessitates greater capacity within the healthcare system to recognize and address the evolving end of life care needs of people who have chronic life-limiting conditions, regardless of where they receive care. It is widely acknowledged that the expertise required for a palliative approach does not lie exclusively with any particular discipline, profession or healthcare sector, and therefore inevitably requires integration into existing care models and systems in partnership with a range of healthcare providers. Our analysis revealed three prominent types of care delivery models for the integration of a palliative approach: *(a) “early” palliative care, (b) integration into generalist practice,* and *(c) disease/condition-specific models for care delivery*. Each of these approaches, which are illustrated in Table 3 with examples and citations, involves an increase in capacity on the part of care providers to provide a palliative approach. However, the way in which this capacity building takes place varies. For example, there are differences in how palliative care specialists are engaged in working with other professionals who do not specialize in palliative care (i.e., generalist care providers or specialist chronic disease management teams). In addition, the different models of care delivery inform how palliative care principles and practices are systematically integrated and contextualized to ensure quality care for different conditions and sectors of care.

Some authors advocate for “early” palliative care whereby palliative care knowledge and expertise is applied upstream with minimal adaptation. This approach remains closely related to traditional palliative care that originated within contexts of cancer care, but with a more upstream orientation. An example is provided in a study by Temel et al. [23] who found that introducing palliative care early on in the diagnosis of people who have metastatic non-small-cell lung cancer resulted in a relative improvement in quality of life and survival. Early palliative care was administered at an outpatient clinic and involved at least monthly visits with specialist palliative care clinicians who provided care according to guidelines with special attention to “assessing physical and psychosocial symptoms, establishing goals of care, assisting with decision making regarding treatment, and coordinating care on the basis of the individual needs of the patients” (p. 734). This model of early palliative care relies on increased and routine involvement of palliative care specialists early on and throughout the illness trajectory. It has been mostly applied to populations of cancer patients for whom palliative care guidelines already exist, and thus minimal adaptation is required.

Another model of care delivery involves widespread system *integration of a palliative approach into generalist practice*, including primary and residential care. A key characteristic is that the models are not uniquely focused on a particular disease but rather applied to particular sectors of healthcare for people who have various life-limiting conditions and comorbidities (e.g., frailty). The GSF is a prime example of an initiative that focuses predominantly on contexts of primary care and residential care. The GSF involves a range of practice support tools, including tools for early identification, assessment, and care planning to address patient and caregiver needs, symptom management, and strategies for ensuring continuity of care and outcomes evaluation [19, 27, 34, 61–63]. In addition to these practice support tools, the GSF facilitates implementation through multidisciplinary engagement and provider education. This approach involves transformation of the healthcare system by building capacity within all members of the healthcare team to address the needs of people with life-limiting conditions, while working collaboratively with palliative care specialists. Studies suggest that the GSF has potential to improve end of life care. Within participating care homes, care providers found that the GSF improved symptom control and team communication, assisted the homes to find helpful external support and expertise, increased staff confidence, and fostered residents’ choice [62]. Badger 2009 [34] similarly found improvements in care homes post-GSF implementation, including statistically significant increases in the proportion of residents who died in the care homes (instead of in a hospital) and those who had an advance care plan; there was also a significant reduction in crisis admissions to hospital. Within primary care, the GSF was shown to improve communication, teamwork, patient identification, assessment, and care planning [27, 64]. However, implementation challenges have also been identified such as practitioner access to training [34]; ensuring consistency and effectiveness in the use of the GSF; and the need for greater emphasis on patient outcomes, economic evaluation, equity, and sustainability [27]. Overall, models for the integration of a palliative approach into generalist practice models involve working with palliative care specialists to develop capacity within the generalist multidisciplinary care team. As noted in Table 3, we found these models have mostly been adopted in primary care and residential care sectors.

Finally, a third approach involves the adaptation of *disease/condition-specific models for care delivery* to integrate palliative care principles and practices within healthcare systems. Practices, such as symptom management, advance care planning, and formulation of goals of care, are increasingly integrated with chronic disease management for people with COPD, CHF, renal disease, neurological conditions, and frailty [9, 10, 16, 53, 65]. This approach is not unique to any particular sector of care, but rather requires coordination across healthcare sectors that are accessed by people with life-limiting conditions at various stages of their illness trajectories. This includes coordination across acute, outpatient, community, and residential care sectors [8, 9, 16, 53]. For example, one study described how COPD patients and their families connect with their chronic care team through information technology and work in partnership with community health nurses who act as mentors to support the patients’ self-management [9]. A key characteristic is that the system of care involves both the expertise of the chronic disease or geriatric specialists as well as specialists in palliative care who collaborate to ensure the full breadth of services required to address the range of needs of patients and their family caregivers. This necessitates ongoing capacity building for a palliative approach through intentional partnership between specialist palliative care and chronic disease management teams [53].

## Conclusion

Findings from our narrative synthesis and thematic analysis suggest that there are key distinctions to be made between a palliative approach and the way in which palliative care has conventionally been enacted as a healthcare service. A particular challenge lies in the use of the term “palliative care” to refer to either a philosophy of care, or a service, or both. For example, in many healthcare systems, there are particular prognostic criteria related to expected length of life for gaining access to palliative care services. However, the application of palliative care as a philosophy does not necessarily require specialized services. The term “palliative approach” is used, in part, to address this tension, where a palliative approach can be enacted by any healthcare professional by adapting palliative care knowledge and expertise to meet the needs of people with chronic life-limiting conditions.

Our synthesis reveals that a palliative approach requires healthcare system change and integration. It is not possible to rely exclusively on expert palliative care services to address the emerging palliative care needs of people who have life-limiting conditions. For example, mechanisms for early identification and advance care planning are needed to ensure that the palliative needs of people who have life-limiting conditions are recognized and addressed early on by all healthcare professionals. This requires healthcare professionals in all sectors of care to acknowledge and address patient and family caregiver needs that arise from the life-limiting nature of the condition, necessitating integration into the education of healthcare professionals. However, there is relatively little mention of healthcare professional education that specifically focuses on a palliative approach, and it is difficult to ascertain to what extent the current palliative care education enables people to provide a palliative approach [66].

Finally, the integration of a palliative approach requires models of healthcare delivery that will facilitate the adaptation and application of palliative care knowledge and expertise in all healthcare sectors and for all chronic life-limiting conditions. To achieve this, there is a need for research to determine which models of care delivery are most appropriate, useful, and cost-effective. This can only be achieved if palliative care knowledge and expertise is extended beyond the domain of palliative specialist services to include the full scope of healthcare services, and if providers are required to address the needs of people who have life-limiting conditions and their families.
